# A detailed molecular analysis of complete Bovine Leukemia Virus genomes isolated from B-cell lymphosarcomas

**DOI:** 10.1186/1297-9716-44-19

**Published:** 2013-03-18

**Authors:** Gonzalo Moratorio, Sabrina Fischer, Sergio Bianchi, Lorena Tomé, Gonzalo Rama, Gonzalo Obal, Federico Carrión, Otto Pritsch, Juan Cristina

**Affiliations:** 1 Unidad de Biofísica de Proteínas, Institut Pasteur de Montevideo, Mataojo 2020, Montevideo 11400, Uruguay; 2 Laboratorio de Virología Molecular, Centro de Investigaciones Nucleares, Facultad de Ciencias, Universidad de la República, Iguá 4225, Montevideo 11400, Uruguay; 3 Departamento Básico de Medicina, Hospital de Clínicas, Facultad de Medicina, Universidad de la República, Montevideo, Uruguay; 4 Departamento de Inmunobiología. Facultad de Medicina, Universidad de la República, Av. General Flores 2125, Montevideo 11800, Uruguay; 5 Laboratorio de Técnicas Nucleares, Facultad de Veterinaria, Universidad de la República, Lasplaces 1550, Montevideo, Uruguay

## Abstract

It is widely accepted that the majority of cancers result from multiple cellular events leading to malignancy after a prolonged period of clinical latency, and that the immune system plays a critical role in the control of cancer progression. Bovine leukemia virus (BLV) is an oncogenic member of the Retroviridae family. Complete genomic sequences of BLV strains isolated from peripheral blood mononuclear cells (PBMC) from cattle have been previously reported. However, a detailed characterization of the complete genome of BLV strains directly isolated from bovine tumors is much needed in order to contribute to the understanding of the mechanisms of leukemogenesis induced by BLV in cattle. In this study, we performed a molecular characterization of BLV complete genomes from bovine B-cell lymphosarcoma isolates. A nucleotide substitution was found in the glucocorticoid response element (GRE) site of the 5' long terminal repeat (5'LTR) of the BLV isolates. All amino acid substitutions in Tax previously found to be related to stimulate high transcriptional activity of 5'LTR were not found in these studies. Amino acid substitutions were found in the nucleocapsid, gp51 and G4 proteins. Premature stop-codons in R3 were observed. Few mutations or amino acid substitutions may be needed to allow BLV provirus to achieve silencing. Substitutions that favor suppression of viral expression in malignant B cells might be a strategy to circumvent effective immune attack.

## Introduction

Bovine leukemia virus (BLV) is a B-lymphotropic oncogenic member of the Retroviridae family that infects cattle worldwide and is the causative agent of enzootic bovine leukosis (EBL), a neoplastic proliferation of B cells [1,2]. BLV infection is characterized by a long period of viral latency and by the absence of viremia. This is thought to be related to the transcriptional repression of viral expression in vivo [3]. Latency is likely a viral strategy to evade the host immune response, thereby allowing tumor development [4,5]. In fact, B lymphocytes harboring an integrated provirus do not produce detectable levels of viral RNA or proteins [6]. Nevertheless, when these cells are isolated and cultured in vitro, a marked increase in viral transcription occurs, suggesting that the provirus is maintained at a repressed stage in vivo [7].

Regarding genome organization, as in all retroviruses, BLV has the gag, pro, pol, env structural genes (from 5′ to 3′ of the genome) required for the production of infectious virions [8]. In addition to these genes, the BLV genome contains an X region located between the env gene and the 3′ long terminal repeat (3′-LTR) [9], as also observed in other Deltaretroviruses [10]. This region contains the open reading frames of four regulatory proteins: the transactivator protein, Tax [11]; the Rex protein, which stabilizes and allows exportation through the cytoplasm of viral RNA [12] and two accessory proteins R3 and G4 whose small open reading frames (ORF) are located in the region between the env gene and the tax/rex genes [13]. Deletion of R3 and G4 genes of BLV in an infectious and tumorigenic BLV molecular clone induced loss of the leukomogenic phenotype and G4 exhibited oncogenic potential both in vivo and in vitro [14,15].

The BLV transcriptional promoter is located in the 5′long terminal repeat (5′-LTR) and is composed of the U3, R and U5 regions. Gene expression is induced at the transcriptional level by the virus-encoded transactivator Tax [16].

Few complete genomic sequences of BLV strains are available in the databases. These sequences are from different sources: peripheral blood mononuclear cells (PBMC) [17], tumor cells, experimentally infected sheep, and cell lines (FLK). The degree of genetic variation among these strains and those directly isolated from bovine B-cell lymphosarcomas remains unknown. For this reason, and in order to contribute to the understanding of the mechanisms of leukemogenesis induced by BLV, we performed a detailed characterization of the complete genome of three BLV isolates from B-cell lymphosarcomas of three cows from different farms, and we compared them with all available and corresponding full length sequences from BLV isolates from other sources.

## Materials and methods

Lymphosarcoma samples were obtained by certified veterinary doctors following appropriate ethical guidelines from national and international veterinary associations. The project was also read and approved by Institut Pasteur-Montevideo, Uruguay.

### Animals

Lymphosarcoma samples were obtained from three dairy cows proven to be infected with BLV by PCR and ELISA (VMRD Inc., Pullman, WA, USA).

### DNA extraction and PCR amplification

DNA samples were extracted from lymphosarcoma tissue and FLK cells (as a control), using the QiAmp DNA Blood Mini kit from QIAGEN, according to the instructions supplied by the manufacturer. PCR amplification of overlapping genome fragments covering the complete genome of BLV was achieved using Phusion DNA Polymerase (New England BioLabs) and specific primers designed for this study (synthesized by Integrated DNA Technologies, Leuven, Belgium and shown in Additional file 1). The location of each amplicon is shown in Additional file 2. Reagents for PCR were from New England BioLabs. The final reaction mixture (50 μL) contained 1x HF buffer, 200 μM dNTP, 200 nM of each primer, and 1 U Taq polymerase. The cycle for the PCR amplification were as follows: 98°C for 30 s, then 30 cycles of denaturation at 98°C for 10 s, annealing at 55–65°C for 30 s, and extension at 72°C for 1–3 min, followed by a final extension at 72°C for 10 min. The PCR reactions were carried out using an Eppendorf Mastercycler Gradient PCR Thermal Cycler.

### Amplicon purification and cloning

Amplicons were resolved by 1% agarose gel electrophoresis, stained with ethidium bromide and purified using QIAquick PCR Purification Kit from QIAGEN, according to instructions from the manufacturers, and cloned into pGEM T- Easy vector (Promega). Electrocompetent XL1-Blue bacteria were transformed by colonies and were expanded and small-scale plasmid purification was performed using the GFX DNA purification kit (GE Healthcare, Piscataway, NJ, USA).

### Sequencing

Both strands of purified plasmids were sequenced in order to avoid discrepancies by using specific and universal T7 or SP6 primers and the Big Dye DNA sequencing kit (Perkin-Elmer) on a 373 DNA sequencer apparatus (Perkin-Elmer). Complete genome sequences were obtained from B-cell lymphosarcomas and deposited in the EMBL database under accession numbers EMBL:HE967301 to EMBL:HE967303 (LS1to LS3). Complete genome sequences were obtained for all available and comparable BLV strains by using All-round Retrieval of Sequence and Annotation (ARSA) at the DNA Data Bank of Japan (DDBJ) [18].

### Sequence alignment

Sequences were aligned using the CLUSTAL W program [19].

### Protein sequences

Protein sequences were obtained by means of in silico translation of nucleotide to amino acid sequences. This was done by using software from the MEGA program [20].

## Results and discussion

### Comparison of the 5′-LTR genome region of BLV strains isolated from lymphosarcomas and other origins

**Figure 1:**
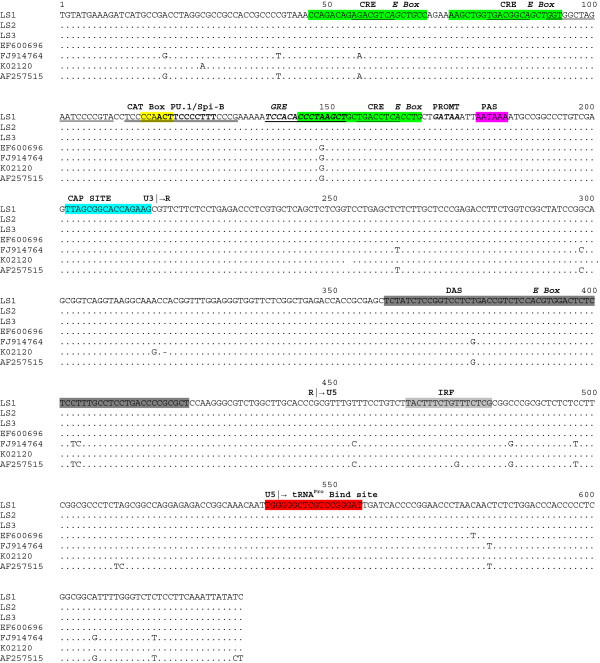
**Alignment of 5**^**′**^**LTR nucleotide sequences of BLV strains.** BLV strains isolated from PBMC cells and previously described are shown by accession number on the left side of the figure. BLV isolates from B-lymphosarcoma tumors (LS1 through LS3) are shown by name. Identity with BLV strain LS1 is indicated by a dot. The U3, R, and U5 regions are indicated on top of the alignment. The three TxRE enhancer regions are shown in green, cyclic AMP- responsive element (CRE) sequences are underlined and E-Box sequences are shown in italics. Binding sites for PU.1/Spi-B are shown in bold. The glucocorticoid responsive element (GRE) binding site is shown in bold, italics and underlined. Nuclear factor κB (NF-κB) binding sites are shown double underlined. The CAT box and GATAA box promoters (PROMT) sequences are indicated in yellow and in bold italics, respectively. The polyadenylation site (PAS) is shown in magenta and the CAP site is shown in light blue. The tRNA proline primer binding sites are shown in red. The downstream activator sequence (DAS) and the interferon regulatory factor (IRF) binding sites are shown in dark and light grey, respectively.

BLV initiates transcription at the U3-R junction of the 5′-LTR induced by Tax protein [16]. Transactivation requires the presence of three 21-bp enhancer elements (called Tax-responsive elements, TxRE) located in the U3 region of the 5′-LTR [21]. Each TxRE contains an octanucleotide core sequence corresponding to an imperfectly conserved cyclic AMP-responsive element (CRE), which binds cellular transcription factors like CRE-binding protein (CREB), CRE-modulator τ isoform (CREMτ), and activating transcription factors 1 and 2 (ATF-1 and ATF-2) [22]. TxRE also contains an E-box sequence, which overlaps each of the three CRE motifs, and binds proteins that belong to the basic helix-loop-helix (bHLH) family of transcription factors, including c-Myc, Max, USF or TFE3 [23]. The U3 region also contains a PU.1/Spi-B binding site [24] and a glucocorticoid responsive-element (GRE) [25]. In addition, BLV expression is regulated by 5′-LTR sequences downstream of the transcription initiation site: a 64-bp downstream activator sequence (DAS) at the 3′ end of the R region [26] and an interferon regulatory factor binding site in the U5 region [27]. A scheme showing the positions of all these elements in BLV 5′-LTR is shown in Figure 1.

Comparison of the 5′-LTR genomic sequences of the three BLV lymphosarcoma isolates (LSI) with all available complete BLV genome sequences, revealed that this genome region is highly conserved (Figure 1). The only significant difference between LSI and those isolated from other cell types, e.g. PBMC or FLK cells, is a base substitution found at position 150 (G to A) in the third enhancer element of this region, at the GRE binding site (Figure 1). It has been previously found that GRE confers responsiveness to glucocorticoids such as dexamethasone in the presence of the Tax transactivator [28]. However, in the absence of Tax, mutation of the GRE significantly decreases basal LTR activity as shown in reporter-based assays [25]. This raises the possibility that this substitution may have allowed a better silencing of viral transcription in the lymphosarcoma strains, as a strategy to avoid recognition by the host immune response [25].

### Comparison of deduced amino acid sequences from structural proteins of BLV LSI with those of other origins

**Figure 2:**
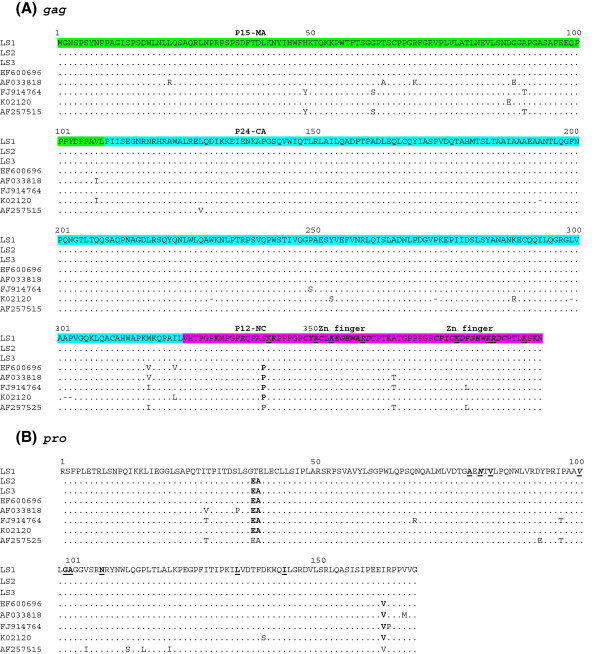
**Alignment of amino acid sequences of Gag and Protease (Pro) proteins of BLV isolates.** In (**A**), alignment of Gag polyprotein sequences is shown. Matrix (p15-MA), capsid (p24-CA) and nucleocapsid (p12-NC) protein sequences are shown in green, light blue and magenta, respectively. Zinc finger domains are shown in bold and italics. NC residues in boldface indicate basic residues. Conserved residues among BLV, HTLV-1 and HTLV-2 are shown bold underlined. In (**B**) alignment of protease (Pro) sequences is shown. Residues proven or predicted to be involved in S2 binding subsites are shown in bold. Residues that appear in HIV-1 drug resistance at the equivalent position in HIV-1 protease [32] are shown in bold underlined. The rest is the same as Figure 1.

In order to detect differences among BLV LSI and isolates from PBMC and other origins, the amino acid sequences of structural proteins encoded by gag, pro, pol, and env genes were aligned. Gag is a polyprotein precursor that is cleaved in the mature virions giving rise to the following: matrix (p15- MA), capsid (p24-CA) and nucleocapsid (p12-NC) proteins (see Figure 2A). NC proteins among all retroviruses share as a major characteristic the presence of a high percentage of basic residues as well as zinc binding domains involved in RNA packaging, both of which are well conserved in all BLV isolates. Indeed, previous studies have shown that substitutions in either basic amino acid residues or zinc finger domains led to a significant reduction in viral RNA packaging [29]. In that sense, a proline to serine (P340S) substitution was observed in the NC protein of all BLV LSI (Figure 2A). This substitution could potentially increase side chain hydrophylicity and be involved in the elimination of the structural restriction related to proline presence. Interactions of NC with RNA sequences, besides those RNA secondary structures of the RNA packaging signal, has been demonstrated for other retroviruses, e.g., murine leukemia virus (MLV) and spleen necrosis virus (SNV). These interactions play an important role in the RNA packaging of these viruses [30,31].

BLV protease (PR) is an aspartic protease with a functional activity involved in gag processing and thus in virion maturation. Previous work proposed a molecular model for BLV PR as well as its substrate specificity, cleavage type sites and inhibitor sensitivity [32]. The comparison of amino acid sequences of PR of BLV LSI with all other sequenced BLV isolates examined in this study is shown in Figure 2B. Only one amino acid substitution (V165I) was found among the BLV lymphosarcoma isolates and is not related to sites previously reported to be involved in BLV PR function via main-chain atoms of peptide substrates or residues predicted to form cleavage subsites [32,33]. Two substitutions can be observed at positions 37–38 in lymphosarcoma BLV isolate LS1, as compared to other genomic sequences including LS2 and 3 isolates (see Figure 2B).

**Figure 3:**
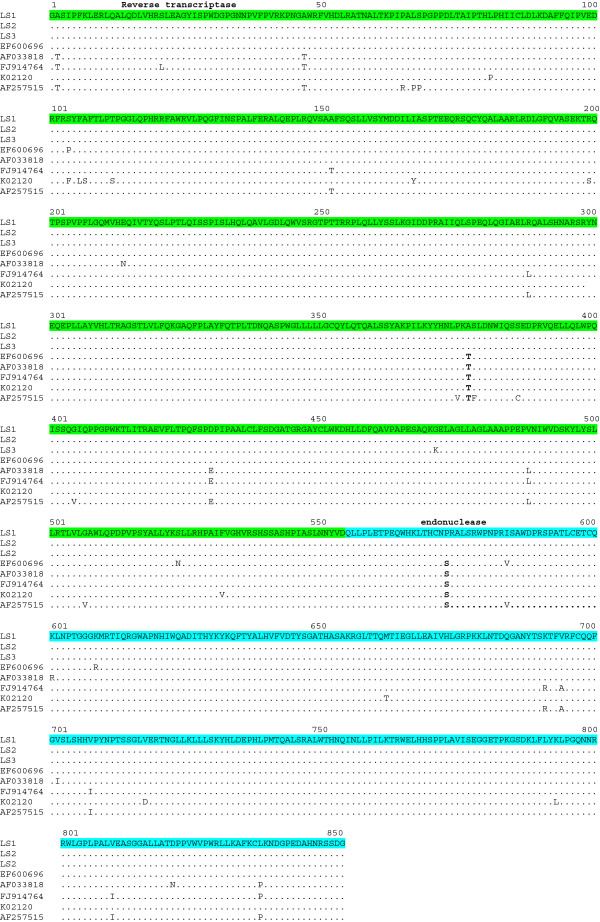
**Alignment of amino acid sequences of Polymerase (Pol) proteins of BLV isolates.** The reverse transcriptase (RT) region is shown in green and the endonuclease region is shown in light blue. The rest is the same as Figure 1.

Two amino acid substitutions can be found in the polymerase precursor of all three BLV isolates, one located in the RT (T378A), the other in the endonuclease region (S573P) (see Figure 3).

This substitution could involve important structural changes, but unfortunately, the structure of BLV polymerase as well as other related Deltaretroviruses, like HTLV-1, is currently unknown.

Further studies will be needed to establish if these substitutions can affect polymerase fidelity or processivity.

The Env protein complex is composed of two component subunits: gp51 surface (SU, N- terminal portion) and gp30 transmembrane (TM, C-terminal portion), which remain associated as a functional trimer with three SU subunits linked by disulphide bonds to a spike of three TM subunits [34]. The gp51protein recognizes and binds to cellular receptors, thereby initiating conformational changes that lead to fusion of viral and cellular membranes by gp30 oligomers [35].

**Figure 4:**
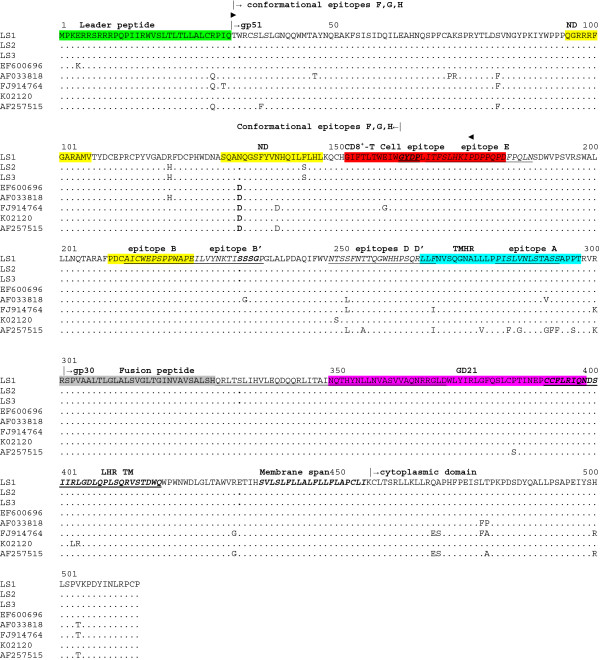
**Alignment of amino acid sequences of Envelope (Env) proteins of BLV isolates.** Amino acid residues corresponding to gp51 (SU) and gp30 (TM) proteins are shown on the top of the alignment. Leader peptide of SU is shown in green. Structural strong turn motif GYPD is shown in bold, italics and double underlined. Conformational epitope region is indicated on top of the alignment. Linear epitopes are shown in italics and underlined [41,42]. Receptor-binding domain (RBD) [43] residues are delimited by two triangles. Second strong turn, SSSG, is shown in bold and italics. Amino acids involved in neutralization domains are shown in yellow. CD8 + −T epitope is shown in red. SU transmembrane hydrophobic region (TMHR) is shown in light blue. TM fusion peptide is shown in light grey and residues believed to span host cell membranes are indicated double lined. BLV leash and α-helical region (LHR) [35] is shown in bold, italics and underlined. Epitope GD21 is shown in magenta. TM membrane-spanning region is shown in bold and italics. The cytoplasmic domain is indicated on top of the alignment. The rest is the same as in Figure 1.

Previous studies have shown that the N-terminal portion of mature gp51 plays an important role in virus infectivity [36]. This region is composed of conformational epitopes F, G and H [37] followed by the structural strong turn GYDP, which is conserved in all oncogenic retroviruses [38]. This motif separates the conformational epitope region from the C-terminal domain of gp51 that contains the linear epitopes A, B, D and E [39] (see Figure 4). Comparison of Env protein of BLV LSI with other previously described isolates, reveals an amino acid substitution in SU conformational epitope region (D134N) in a location previously shown to be related to neutralization [39] (Figure 4). However, this substitution has been previously described as a signature of BLV strains circulating in Uruguay, and it is not specific for LS samples [40].

### Comparison of deduced amino acid sequences from non-structural proteins of BLV LSI and other origins

**Figure 5:**
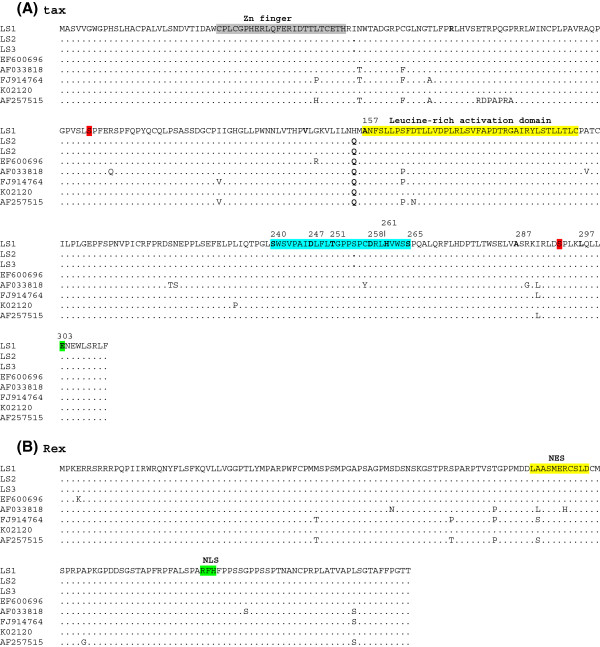
**Alignment of amino acid sequences of Tax and Rex proteins of BLV isolates.** In (**A**) the alignment of Tax sequences is shown. The region between amino acids 240 and 265, in which missense mutations influence the transactivation activity of the Tax protein [46] is shown in light blue. A putative zinc finger domain is shown in grey, a leucine-rich activation domain in yellow and sites of phosphorylation are indicated in red. Position 303, where a previously described substitution E303K gave rise to a replication-deficient virus [47] is shown in green. Positions where substitutions have been previously reported to have an effect on transactivation activity are shown in bold. In (**B**) alignment of Rex sequences is shown. Nuclear export signal (NES) is shown in yellow and the nuclear localization signal (NLS) is shown in green. The rest is the same as Figure 1.

Previous studies on the functional domains of the BLV Tax protein have identified a putative zinc finger motif (amino acids 30 to 53), a transactivating domain (amino acids 157 to 197) and two phosphorylation sites (amino acids 106 and 293) [44] (see Figure 5A). A series of BLV Tax mutants with strikingly more ability to stimulate BLV LTR-directed transcription in comparison with wild-type Tax have been previously described. All these mutants have substitutions between amino acid 240 and 265 [45]. Amino acid changes previously related to higher transcriptional activity as well as changes in the previously described phosphorylation sites were not observed in all the BLV isolates examined in the present study (see Figure 5A). Only one substitution was found in strain LS1 outside the leucine-rich activation domain (Figure 5A). Whether this amino acid substitution has an effect on BLV transcription is currently unknown.

Previous studies revealed that silencing is critical for tumor progression and distinct genetic and epigenetic mechanisms were identified for complete suppression of BLV Tax expression.

Conservation of sites involved in suppression of viral expression may be an important factor for the uncontrolled proliferation of BLV-infected tumor cells [5].

The Rex proteins of Deltaretroviruses act to facilitate the export of intron-containing viral RNA [48]. The Rex proteins shuttle between nucleus and cytoplasm using the nuclear localization signal (NLS) and nuclear export signal (NES) (see Figure 5B). No significant substitutions were found in Rex proteins of all BLV strains enrolled in this study.

**Figure 6:**
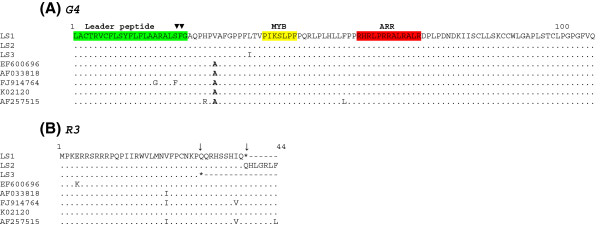
**Alignment of amino acid sequences of accessory proteins R3 and G4 of BLV isolates.** In (**A**) alignment of G4 sequences is shown. The leader peptide is shown in green, and the two putative cellular protease cleavage sites are indicated by inverted triangles on top of the alignment. The myb-like motif (MYB) and the arginine-rich nucleus targeting RNA-binding region (ARR) are shown in red. In (**B**) alignment of R3 sequences are shown. Termination codons are shown by an asterisk and indicated by an arrow.

G4 protein amino acid sequence includes an amino-terminal stretch of hydrophobic residues (amino acids 1 to 24) followed by potential proteolytic cleavage sites and an arginine-rich region (amino acids 58 to 72) located in the middle of the protein [13] (see Figure 6A). This latter region is required for the interaction of G4 with cellular protein farnesyl pyrophosphate synthetase (FPPS), (phosphorylation) [49]. The biological relevance of G4-FPPS interaction has been previously demonstrated in cellular transformation. Mutations in the arginine-rich α-helix of G4 abrogate primary cell immortalization and induction of tumors in nude mice [49]. Therefore, disruption of the interaction between G4 and FPPS could interfere with the oncogenic process.

No amino acid substitutions were found in the arginine-rich α-helix of G4 protein of the previously sequenced BLV isolates examined in this work (see Figure 6A). Nevertheless, an amino acid substitution (A29V) can be observed in G4 of all BLV LSI.

Interestingly, premature stop codons were observed in R3 of two of the three LS BLV isolates (Figure 6B). Previous studies on BLV infection using sheep provide insight on the molecular genetic and epigenetic modulation of viral expression [50]. These studies show that the deletion of the region that expands from the end of the *env* gene to the splice acceptor site of the *tax/rex* mRNA does not impair infectivity [21]. These sequences correspond to the third and second exons of R3 and G4, respectively, revealing that these sequences may not be essential for infectivity in vivo. Although previous studies have shown that deletions in R3/G4 interferes with the efficiency of BLV propagation and restricts pathogenesis [14,15,46,49], another study has shown that one out of 20 sheep infected with a R3/G4 mutant developed a lymphoma after 7.5 years of latency, suggesting that the deleted sequences may not be strictly required for pathogenesis [51]. Further studies will be needed to address the biological significance of these findings in studies using the cow as a model for BLV infection.

In summary, although the genome of BLV is highly conserved in our isolates and in isolates from other sources previously described, variations can be observed in some genome regions. It is thought that silencing of viral expression is a multi-step process leading to the uncontrolled growth of a transformed B-cell clone and the onset of disease [5] and is critical for tumor progression and proliferation of BLV-infected tumor cells [5], as well as escaping recognition by the host immune response [4]. In that sense, the substitution found in the GRE site of the 5′LTR of all BLV strains isolated from the lymphosarcomas might contribute to these factors, since previous studies have shown that substitutions in GRE site significantly reduces basal LTR transcription activity [52] (see Figure 1). Moreover, all amino acid substitutions in Tax previously found to be related to stimulate high transcriptional activity of 5′LTR were not found in this study (see Figure 5A). Genetic and epigenetic mechanisms have been recently proposed for BLV suppression of viral gene expression [53]. The results of the present report, using full-length genome sequences, suggest that point mutations along the whole genome may also be needed to allow BLV provirus to achieve silencing.

## Competing interests

All authors declare that they have no competing interests.

## Authors’ contributions

GM and JC conceived the study; GM and SF have made substantial contributions to the acquisition of the data and the analysis. GO, LT, GR, FC and SB made substantial contribution to the interpretation of the data. OP has made substantial contribution to interpretation of data and has been involved in critically revising the manuscript for important intellectual content and made important contributions to the interpretation and discussion of the results found in this work. JC and GM participated in the different analysis and wrote the paper. All authors read and approved the final manuscript.

## Supplementary Material

Additional file 1 **Primers used for amplification and sequencing of the BLV genome.** List of primers used for amplification and sequencing of full-length genome sequences of the BLV strains. Click here for file

Additional file 2 **Strategy for amplification and sequencing of full-length BLV genomes.** A scheme shows the strategy for amplifying the BLV genome in two long PCRs and the strategy used for sequencing full-length BLV genomes. A scheme of BLV genome is shown on top of the figure and the relative position of LTR and BLV proteins in the BLV genome can be seen by the bar undelying the genome scheme. BLV was amplified in two long PCR shown in yellow using appropriate primers shown bellow. The position of the primers used for sequencing is also shown on the bottom of the figure. Click here for file
