# Supplementary material for: A detailed molecular analysis of complete Bovine Leukemia Virus genomes isolated from B-cell lymphosarcomas
Source: Vet Res. 2013 Mar 18;44(1):19. doi: 10.1186/1297-9716-44-19 (PMC3618307; doi:10.1186/1297-9716-44-19)
Supplement: Additional file 1 — Primers used for amplification and sequencing of the BLV genome. List of primers used for amplification and sequencing of full-length genome sequences of the BLV strains. [file 1297-9716-44-19-S1.doc]

**Additional File 1 Primers used for amplification and sequencing of the BLV genome.**

| **Primer** | S**equence** | **Nucleotides** | **Polarity** | | |
| --- | --- | --- | --- | --- | --- |
| **Geno 01F** | tgtatgaaagatcatgccgac | 1-21 | | Sense |  |
| **Geno 02R** | agcctttgcgcgctttgvvgag | 692-713 | | Antisense |  |
| **Geno 03F** | attgatcaccccggaacccta | 552-572 | | Sense |  |
| **Geno 04R** | ggagatttttccaggcctgaagcc | 1308-1331 | | Antisense |  |
| **Geno 05F** | atgaccagcctaacggcagca | 1168-1188 | | Sense |  |
| **Geno 06R** | ctaattcggtcccactaagag | 1865-1885 | | Antisense |  |
| **Geno 07F** | ttcccattggaaacgagactg | 1771-1791 | | Sense |  |
| **Geno 08R** | atttggtttccgtaccgggaa | 2442-2462 | | Antisense |  |
| **Geno 09F** | cgagccacattggattagaac | 2288-2308 | | Sense |  |
| **Geno 10R** | aattggggatgagatctgcaa | 3000-3020 | | Antisense |  |
| **Geno11F** | ggtcacaatgttatcaagccc | 2851-2871 | | Sense |  |
| **Geno 12R** | aatatgctcctcgtcctgtag | 3655-3675 | | Antisense |  |
| **Geno 13F** | aattgtggccccagatttcct | 3514-3534 | | Sense |  |
| **Geno 14R**  **GP51F** | tgggttatatcggcctgccaa  atgccyaaagaacgacgg | 4193-4213  4873-4849 | | Antisense  Sense |  |
| **Geno 15F** | ttgtctcgatggccgaaccca | 4053-4073 | | Sense |  |
| **Geno 16R**  **GP51R** | ggagcatctccaagtctggat  cgacgggactaggtctgaccc | 4922-4942  5724-5744 | | Antisense  Antisense |  |
| **Geno 17F**  **TMR**  **TAXF** | agctcctccggcaggctccc  tcaagggcagggtcggagg  caagtgttgttggttgggggcc | 6228-6247  6361-6379  7259-7280 | | Sense  Antisense  Sense |  |
| **Geno 18R** | ggcaccaggcatcgatggtg | 7328-7347 | | Antisense |  |
| **Geno 19F**  **TAXR** | attctacccctaggcgagcc  ccaagcttcaaaaaaggcgggagagc | 7853-7878  8156-8177 | | Sense  Antisense |  |
| **Geno 20R** | ctctcctggccgctagagggc | 8700-8720 | | Antisense |  |
|  |  |  | |  |  |
|  |  |  | |  |  |
|  |  |  | |  |  |
