# Supplementary figures and images for: A detailed molecular analysis of complete Bovine Leukemia Virus genomes isolated from B-cell lymphosarcomas
Source: Vet Res. 2013 Mar 18;44(1):19. doi: 10.1186/1297-9716-44-19 (PMC3618307; doi:10.1186/1297-9716-44-19)

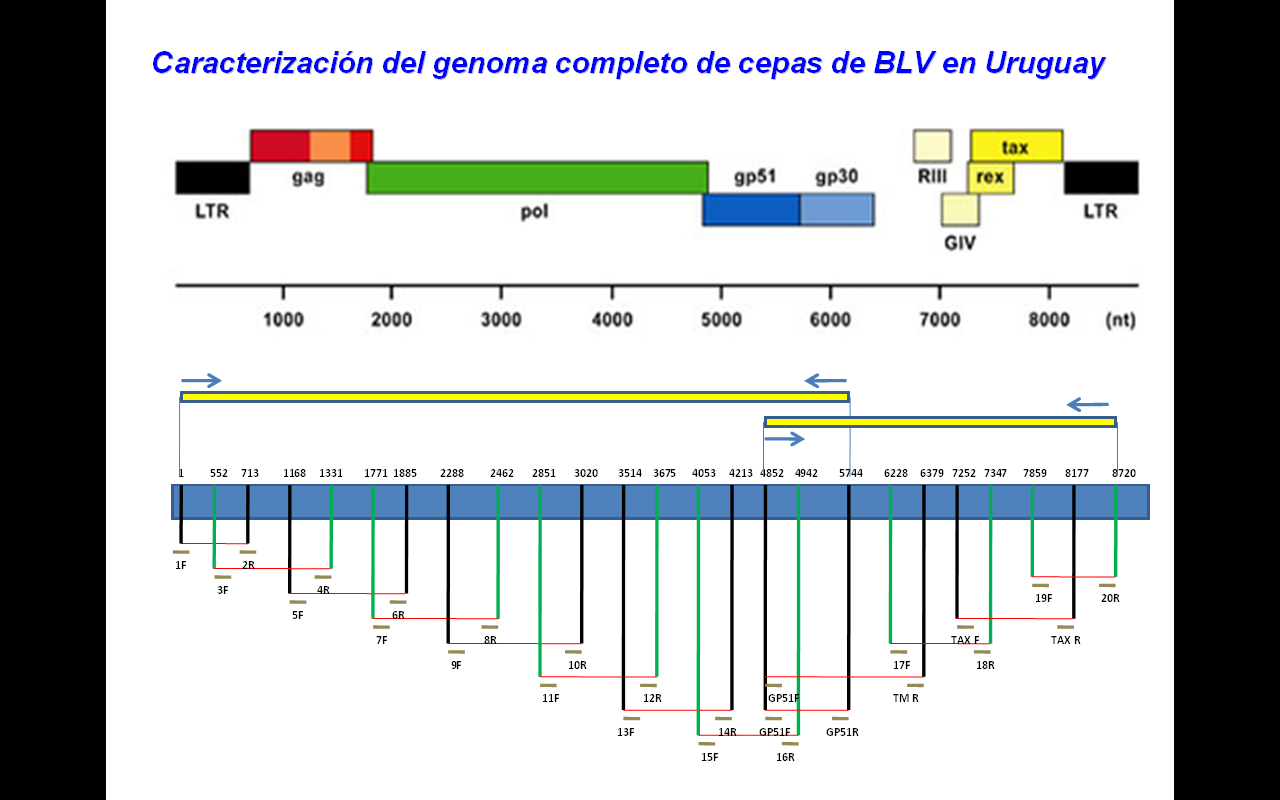


**Additional File 2 Strategy for amplification and sequencing of full-length BLV genomes.**

Supplement: Additional file 2 — Strategy for amplification and sequencing of full-length BLV genomes. A scheme shows the strategy for amplifying the BLV genome in two long PCRs and the strategy used for sequencing full-length BLV genomes. A scheme of BLV genome is shown on top of the figure and the relative position of LTR and BLV proteins in the BLV genome can be seen by the bar undelying the genome scheme. BLV was amplified in two long PCR shown in yellow using appropriate primers shown bellow. The position of the primers used for sequencing is also shown on the bottom of the figure. [file 1297-9716-44-19-S2.doc]
